# First report of the invasive mosquito species *Aedes koreicus* in the Swiss-Italian border region

**DOI:** 10.1186/s13071-015-1010-3

**Published:** 2015-07-30

**Authors:** Tobias Suter, Eleonora Flacio, Begoña Feijoó Fariña, Lukas Engeler, Mauro Tonolla, Pie Müller

**Affiliations:** Department of Epidemiology and Public Health, Swiss Tropical and Public Health Institute, Socinstrasse 57, PO Box, 4002, Basel, Switzerland; University of Basel, Petersplatz 1, 4003, Basel, Switzerland; Gruppo cantonale di Lavoro Zanzare, Via Castello, 6952, Canobbio, Switzerland; Laboratory of Applied Microbiology, University of Applied Sciences and Arts of Southern Switzerland, Via Mirasole 22a, 6501, Bellinzona, Switzerland

**Keywords:** Invasive species, Mosquito surveillance, Mosquito diagnostics, MALDI-TOF MS

## Abstract

**Background:**

In 2012 and 2013, an entomological survey of *Aedes albopictus*, the Asian tiger mosquito, was carried out in the border region of southern Switzerland and northern Italy, using ovitraps. In July 2013, besides *A. albopictus* already known to the region several unusual eggs were recovered.

**Findings:**

A total of 548 seemingly different eggs were found within three communities: Chiasso (Switzerland), and Como and Brunate (Italy). Proteomic diagnostics based on matrix-assisted laser desorption/ionization mass-spectrometry (MALDI-TOF MS) and morphological identification of one reared adult revealed the presence of at least 18 *A. (Finlaya) koreicus* (Edwards, 1917) specimens. *A. koreicus* is a species native to Southeast Asia and is competent to transmit Japanese encephalitis and potentially other arboviruses, as well as the dog heartworm *Dirofilaria immitis*. While new to Switzerland, this invasive species has previously been reported from Belgium, north-eastern Italy and European Russia.

**Conclusions:**

This is the first report of the introduction of this exotic mosquito species into Switzerland and Lombardy, Italy, suggesting the range of *A. koreicus* is expanding in Central Europe. As *A. koreicus* is competent to vector pathogens its establishment imposes a risk to public and veterinary health. From a technical point of view, the presence of *A. koreicus* alongside *A. albopictus* requires careful analysis and reliable diagnostics. As a diagnostic tool the use of the recently developed MALDI-TOF MS approach has proofed to be a very useful approach, particularly since hatching rates of *A. koreicus* seem to be low, making identification by classic morphology difficult, if not impossible.

## Background

After the introduction and establishment of the Asian tiger mosquito, *Aedes albopictus* (Skuse), in the Swiss Canton of Ticino in 2003, an entomological surveillance and control programme was instigated by the canton’s mosquito working group, Gruppo cantonale di Lavoro Zanzare (GLZ) [1]. GLZ uses ovitraps for the surveillance of *A. albopictus* as a basis for interventions in urbanised areas where the mosquito has become endemic [2]. Intervention is chiefly based on larval source reduction through public awareness campaigns that include the distribution of leaflets, a hotline and a website, and by larviciding using diflubenzuron and *Bacillus thuringiensis* var. *israeliensis (Bti)*, targeting small water containers (i.e. < 200 L) such as catch basins, plant saucers, drums, buckets, tarpaulins, tyres and bathtubs [2]. Larger water bodies were inspected for the presence of larvae using standard dippers (model 1132, BioQuip Products, Rancho Dominguez, USA). If immatures were present, the breeding sites were also included in the control approach.

**Fig. 1:**
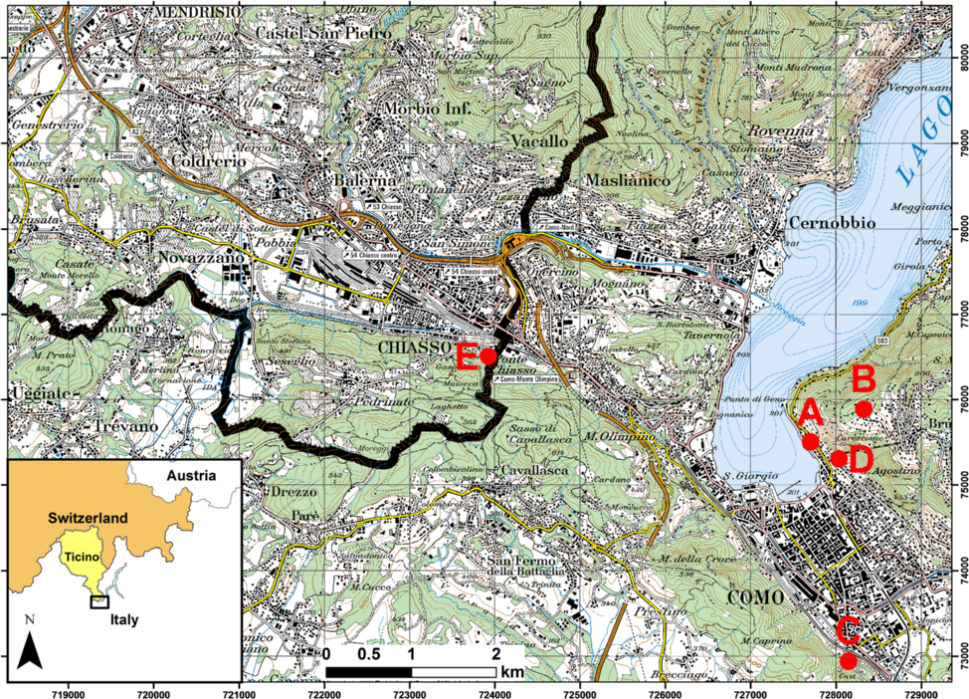
Locations of ovitraps positive for *Aedes koreicus* across the Swiss-Italian border in 2013. Details for the locations and identified eggs are given in Table 1. The numbers at the border indicate the Swiss grid km coordinates. The thick black line represents the Swiss-Italian border. Map layers were purchased from the Swiss Federal Office of Topography

In 2012 and 2013, as part of a research project the mosquito survey was expanded across the Swiss-Italian border, including the Mendrisiotto district (Switzerland) and the northern part of the Lombardy (Italy). In total, the study area covered a surface area of 118 km^2^, 65 km^2^ on the Italian and 53 km^2^ on the Swiss side of the border (Fig. 1). Using the ArcGIS version 10.0 (ESRI Inc., USA) geographic information system software a grid with cells measuring 250 m by 250 m was superimposed over the study area. Seventy grid cells were then randomly selected in both countries using the “sample()” function in the statistical software R version 2.11 [3]. Within each selected grid cell, relative *A. albopictus* densities were measured by placing two ovitraps, set apart at a distance of at least 50 m to avoid interference in attraction. All traps were geo-referenced with a nüvi 1390 (Garmin, Switzerland). Field surveys were carried out from July to November in 2012 and May to November in 2013.

Ovitraps comprised 1.5 L, black plastic flower pots (Ramona Hydro, Luwasa, Switzerland) filled with 1.2 L tap water. Three equally spaced holes were drilled 2 cm below the rim to prevent the traps from being flooded by rain. A wooden slat made of untreated beech wood, measuring 20 cm × 2.5 cm × 0.5 cm, was placed inside the pot so that it was partially submerged and partially stuck out of the water. To prevent the ovitraps from becoming potential larval habitats, larvicide granules of *Bti* (VectoBac®, Valent BioSciences, USA) were added. The slats, water and *Bti* were replaced biweekly. The slats were individually labeled and wrapped in cling film for transport and preservation.

## Findings

By the end of July 2013, a total of 19,328 mosquito eggs from 280 ovitraps were collected across the study area. For five traps (Table 1 and Fig. 1) placed within the communities of Chiasso, (Switzerland), Como and Brunate (Italy), routine egg inspections in the laboratory revealed unusual eggs that were very similar to *A. albopictus* and yet appeared different to the trained eye. Though morphologically not entirely distinct, the eggs seemed to be somewhat more elongated and pointed. In total, we noticed 548 such unusual eggs.

**Table 1:** Field-caught mosquito eggs identified as *Aedes koreicus* across the Swiss-Italian border in 2013

Trap^1^	Position	Community	Total egg number	Suspected as *A. koreicus*	Confirmed as *A. koreicus*
A	45.819 N, 9.082E	Brunate (Italy)	249	131	3
B	45.822 N, 9.090E		47	21	2
C	45.796 N, 9.087E	Como (Italy)	273	125	3
D	45.817 N, 9.086E		402	201	7
E	45.829 N, 9.033E	Chiasso (Switzerland)	92	70	3^2^

^1^The letters correspond with Figure 1

^2^All eggs from trap E remaining after the MALDI-TOF MS analysis were incubated in tap water. One female imago emerged

In the laboratory, the wooden slats collected from the ovitraps in Switzerland were incubated in tap water in an attempt to hatch the eggs. The water was allowed to settle for 24 h before use. The eggs were incubated for 7 days in a climate-controlled chamber (KBWF 720 E5.2, Binder GmbH, Germany) at a temperature of 28 °C, a relative humidity of 70 % and a light:dark cycle of 16:8 h. Unfortunately, only one larva hatched from which a female imago emerged. The specimen was morphologically identified as *A. (Finlaya) koreicus* (Edwards, 1917) using the taxonomic key of Ree [4], and later confirmed by Francis Schaffner (pers. comm.), a renowned expert in mosquito taxonomy. The identified female corresponded to the morphological form known from the South Korean volcanic island Jeju-do [5]. This form has also recently been reported from Belgium [6] and north-eastern Italy [7].

Given the low hatching rate, a subsample of two to six eggs per slat (Table 1) were tested by matrix-assisted laser desorption/ionization mass-spectrometry (MALDI-TOF MS) in combination with a validated database, curated at Mabritec SA (Riehen, Switzerland) [8, 9]*.* Eggs selected for analysis were deposited close to each other and at a maximum distance from the eggs suspected to be *A. albopictus*. For the MALDI-TOF MS analysis the eggs were carefully detached from the slat with a brush and then prepared and processed as described by Schaffner et al. [9]. All selected egg specimens were determined as *A. koreicus* (n = 17, Table 1).

## Discussion and conclusions

*A. koreicus* was originally found in Korea, Japan, China and Eastern Russia [5]. Its introduction has previously been reported from Belgium in 2008 [6], north-eastern Italy in 2011 [7] and European Russia in 2013 [10]. Meanwhile, the mosquito species has successfully established local populations in Belgium and north-eastern Italy [6, 11], confirming its ability to colonise new areas in temperate regions. Although in nature *A. koreicus* breeds in rock pools and tree holes, it successfully utilises artificial breeding sites in more urban environments, very much like other invasive *Aedes* species, including *A. albopictus* and *A. japonicus* (Theobald). Host seeking females may feed on humans and domestic animals both during the day and at night [12]. At the end of the annual season when daylight becomes shorter, *A. koreicus*, like other members of the *Aedes* group, deposits eggs that are dormant and more resistant to desiccation and cold temperatures than eggs laid during the season. Dormant eggs catalyse the mosquito’s passive distribution to new areas [13]. Capelli et al. [7] showed that *A. koreicus* dormant egg stages are even more resistant to cold temperatures compared to *A. albopictus*; and hence this species has the potential to colonise a much wider area of Switzerland and other parts of Europe.

Here, for the first time, *A. koreicus* was found in Switzerland and in the neighbouring Italian Lombardy region. It was not detected during the surveillance activities in 2012, but only in the 2013 summer season. As no *A. koreicus* eggs were found in 2012, we assume that this species has been introduced de novo although we cannot exclude that it was missed during the 2012 survey. While this is the first report of the introduction of this exotic mosquito species into Switzerland and the neighbouring Italian region, it suggests that the range of *A. koreicus* is generally expanding in Central Europe. Continued surveillance will show if this new invasive mosquito has established a local population and is gaining further ground.

Following the example of other invasive mosquito species, trade with used tyres and domestic plants has been suggested to be the route of entry [14–16]. As the single imaginal specimen from this study corresponds to the morphological form found in Belgium [6] and north-eastern Italy [7], these introductions might all be linked to the same mode of introduction, either at the regional or global level, or even both.

*A. koreicus* was suspected as a vector of Japanese encephalitis virus [17, 18] and of the dog heartworm *Dirofilaria immitis* [19]. However, its full vector status is currently not resolved and requires further vector competence and field studies.

While the public health relevance of *A. koreicus* is still subject to debate, its presence in the southern part of Switzerland complicates the routine surveillance of *A. albopictus*. It is not possible to distinguish the eggs unambiguously by morphology through a stereo microscope. The eggs need either to be hatched out and reared to late developmental stages for species identification or analysed by technically more sophisticated tools such as DNA sequencing [20] or the MALDI-TOF MS approach applied here [8]. In this study, the full cost per MALDI-TOF MS sample was 10 Euros. This is considerably lower than for PCR. The preparation of the samples is very simple and only takes a few minutes and the analysis itself takes only a few seconds. We appreciate that in a future study more specimens could be processed to gain a more complete picture of the situation.

The expansion and establishment of this, and potentially other, invasive mosquito species in Switzerland and Europe, as a whole, has to be observed carefully. It is important to implement methods that are able to detect a range of invasive species on a routine basis. As such, MALDI-TOF MS is a very useful tool for the identification of mosquito eggs, significantly simplifying surveillance and species-targeted interventions.
